# Influence of Diet and Tobacco Smoking on Pancreatic Cancer Incidence in Poland in 1960–2008

**DOI:** 10.1155/2012/682156

**Published:** 2012-12-18

**Authors:** Mirosław Jarosz, Włodzimierz Sekuła, Ewa Rychlik

**Affiliations:** National Food and Nutrition Institute, Powsińska Street 61/63, 02-903 Warsaw, Poland

## Abstract

The aim of the study was to investigate the relationship between pancreatic cancer incidence and selected dietary factors, alcohol consumption, and tobacco smoking in Poland in 1960–2008. Data on pancreatic cancer morbidity were derived from the National Cancer Registry and on food consumption from the national food balance sheets. In 1960–1989 correlations were found between pancreatic cancer incidence rates and energy (0.60 for males and 0.57 for females), cholesterol (0.87 and 0.80), fibre (−0.84 and −0.89) and folate (−0.45 and −0.49) intake, the consumption of total fats (0.94 and 0.91), animal fats (0,90 and 0,82), sugar (0.88 and 0.87), cereals (−0.93 and −0.91), and alcohol (0.86 and 0.82). In 1990–2008 morbidity correlated with the consumption of red meat (0.67 and 0.48), poultry (−0.88 and −0.57), and fruit (−0.62 and −0.50). Correlation with tobacco smoking was observed in the whole studied period (0.55 and 0.44). Increased incidence of pancreatic cancer in 1960–1995 was probably related to adverse dietary patterns up to 1989, especially high consumption of fats, sugar, and alcohol. Further positive changes in the diet such as lowering red meat consumption and increasing fruit consumption could influence incidence reduction in recent years. Also changes in tobacco smoking could affect the morbidity.

## 1. Introduction

Pancreatic cancer is one of the cancers of limited occurrence. It mostly concerns high-income countries, where it occurs three times as often as in middle- and low-income countries [1].

In its early stage symptoms are barely observable, and, when diagnosed, the disease is usually in an advanced stage [1, 2]. Therefore survival rates are low; 90% of patients die within 12 months from the moment of diagnosis [3]. In Europe pancreatic cancer is the 6th leading cause of cancer death [4]. In Poland it is ranked 7th among men and 6th among women in the list of cancers that are most frequent causes of death [5].

In that situation understanding the etiology and identifying the risk factors is essential for the primary prevention of this disease. Potentially modifiable risk factors include tobacco smoking, obesity, and diabetes mellitus [3, 6]. The extent to which diet affects pancreatic cancer risk is still unclear. Among nutritional factors that can reduce or increase the risk of pancreatic cancer, the following are listed most often: consumption of food containing folate, fruit, red meat, cereals, and fat [1]. Another important factor is probably chronic pancreatitis, the main cause of which is alcohol consumption [7, 8].

Prevention could play an important role in reducing pancreatic cancer mortality, and an important question is whether diet interventions can lower the risk. Only long-term studies covering several dozens of years, examining how dietary pattern changes affect incidence rates, can ensure a chance to make an objective assessment of this matter. 

The present paper is an attempt to investigate the relationship between the changes in pancreatic cancer incidence rates and selected dietary factors, alcohol consumption, and tobacco smoking in the period from 1960 to 2008.

## 2. Methods

Data on pancreatic cancer incidence rates were derived from the National Cancer Registry administrated by the Maria Skłodowska-Curie Memorial Cancer Center and Institute of Oncology in Warsaw [5, 9]. They showed age-standardized incidence rates for men and women covering years between 1960 and 2008 excepting 1984, 1986, 1997, and 1998, for which no such data were available. “Standard global population” was taken as a standard.

The source of information on the dietary pattern in the same period was the database established by the National Food and Nutritional Institute [10–12]. This database covers data derived from the national food balance sheets and shows major food groups quantities available for consumption per capita/year. The population averages for consumption do not give separate values for men and women, nor actual consumption at the individual level. The methodology of the food balance sheets preparation is fairly harmonized internationally due to the efforts of the FAO [13]. The data on food group consumption are converted into energy and nutrients intake (per person/day) with the use of the national food composition tables [14]. 

Information on alcohol consumption (as total expressed in pure alcohol and including consumption of major types of alcoholic beverages and their alcohol content) and tobacco smoking showing number of cigarettes per capita/year was derived from national statistical year books [15].

The study was focused on identification and measurements of the relationship between pancreatic cancer incidence rates and variables related to dietary pattern represented by the intake of energy, folate, cholesterol and dietary fibre and the consumption of red meat, poultry, fruit, total and animal fats, cereals, and sugar. Data on red meat consumption covering individual years were available from 1974 onwards while earlier information in this regard included 1960 and 1970 only. There was also lack of data on poultry consumption in 1961–69, 1971–74, and 1976-77. Moreover possible influence of alcohol consumption and cigarette smoking on pancreatic cancer morbidity was analyzed.

The relationship between the above risk factors and pancreatic cancer incidence rates was analyzed for the whole period of 1960–2008 and for two subperiods: 1960–1989 and 1990–2008, due to the courses and dynamics of dietary pattern changes before and after the economic transformation.

Spearman's rank correlation coefficients (*r*) were estimated as a measure of the relationship between pancreatic cancer incidence rates and selected parameters.

## 3. Results

Pancreatic cancer is more frequent among men than among women. In 2008 in Poland a standardized incidence rate amounted to 6.1/100 thousand for males and 3.9/100 thousand for females [5]. Between 1960 and 1995 pancreatic cancer incidence among men increased from 1.8/100 thousand to 8.6/100 thousand and from 1.1/100 thousand to 5.0/100 thousand among women [5, 9]. Since then, a favourable downward trend has been observed, which is more evident among men.

Correlation coefficient (*r*) and *P* values between dietary factors or tobacco smoking and pancreatic cancer incidence rates are presented in Table 1. 

**Figure 1:**
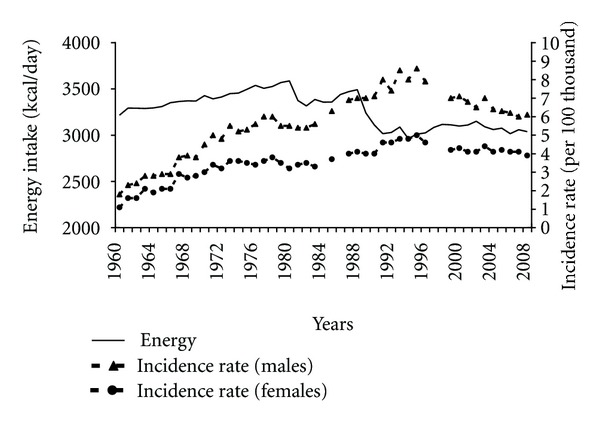
Energy intake and pancreatic cancer morbidity 1960–2008.

One of the factors increasing the risk of pancreatic cancer is excessive adipose tissue, accrued, among others, as a result of a diet with excessive energy content. Up to 1989 the energy content of a typical Polish diet was relatively high (Figure 1) and correlated positively with the increasing incidence of pancreatic cancer (correlation coefficient of 0.60 among males and 0.57 among females). Reduced energy intake on the turn of the 80s and 90s of the 20th century may have positively influenced the reduced incidence although no correlation between both variables had been observed.

**Figure 2:**
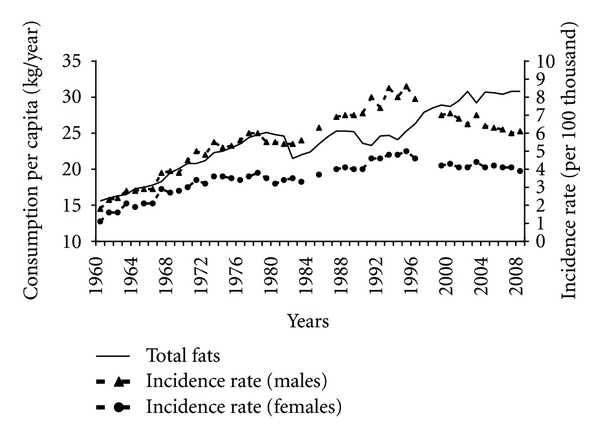
Total fats consumption and pancreatic cancer morbidity 1960–2008.

**Figure 3:**
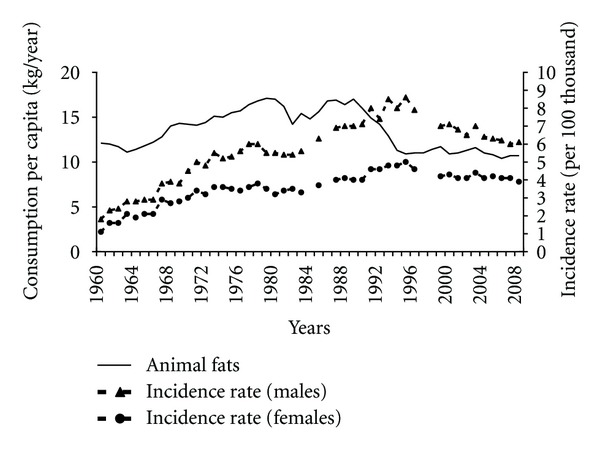
Animal fats consumption and pancreatic cancer morbidity 1960–2008.

Consumption of fats, including animal fats, may have been related to pancreatic cancer incidence. Since 1960s the increased overall consumption of fats took place (Figure 2). Between 1960 and 1989 the correlation between the consumption of these products and the incidence was almost complete (0.94 among males and 0.91 among females). Incidence also correlated positively with the escalating consumption of animal fats (0.90 among males and 0.82 among females). After the transformation the structure of fats consumption changed and the share of animal fats in a diet significantly fell (Figure 3).

**Figure 4:**
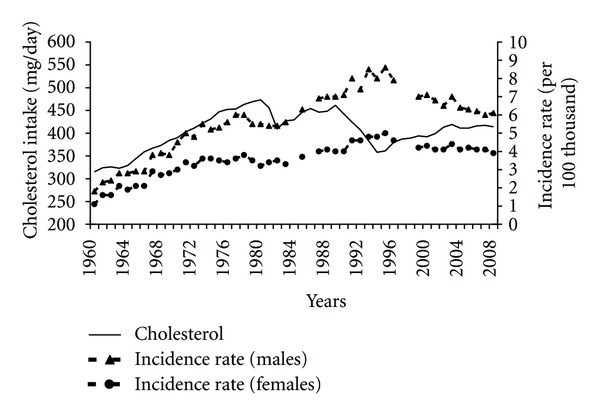
Cholesterol intake and pancreatic cancer morbidity 1960–2008.

In the period after the transformation changes concerning the cholesterol content in a diet also took place (Figure 4). Between 1960 and 1989 the cholesterol intake correlated positively with incidence rates (0.87 among males and 0.80 among females). Reduced cholesterol intake in later years may have been one of the factors contributing to the reduced incidence of pancreatic cancer, despite the lack of positive correlation between these variables.

**Figure 5:**
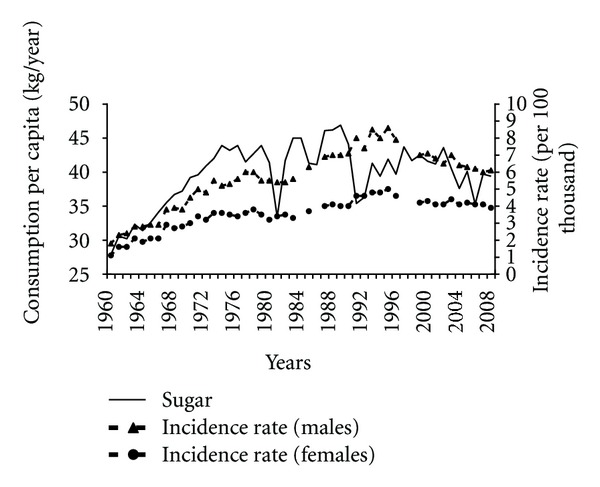
Sugar consumption and pancreatic cancer morbidity 1960–2008.

Before 1995 one of the unfavourable factors contributing to the increased pancreatic cancer incidence had probably been the sugar consumption, which had initially been growing to remain at a high level (Figure 5). In the years 1960–1989 the sugar consumption correlated positively with incidence rates (0.88 among males and 0.87 among females). Although in later years no correlation between these variables was found, we cannot rule out that the reduced sugar consumption may have beneficially influenced the incidence trends.

**Figure 6:**
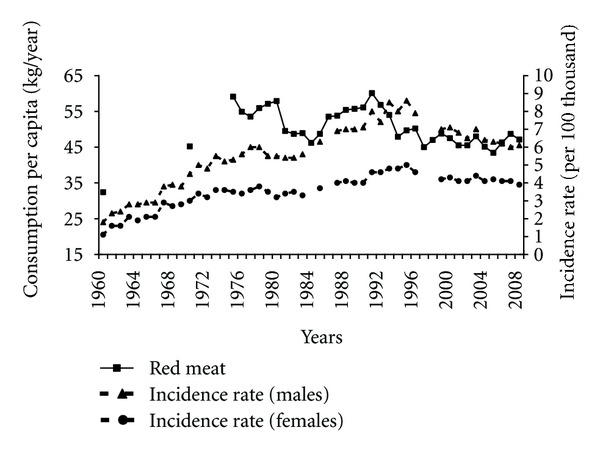
Red meat consumption and pancreatic cancer morbidity 1960–2008.

The consumption of red meat is a factor increasing pancreatic cancer risk. After 1960 the red meat consumption was significantly growing and although later on some fluctuations were observable it remained high (Figure 6). In spite of the lack of correlation, it cannot be ruled out that its high consumption had a certain impact on the morbidity in the years 1960–1989. Between 1991 and 1997 the red meat consumption decreased and stabilized on a lower level than before. This might have been one of the reasons for the reduction in incidence after 1995, which is demonstrated by a positive correlation between these variables (0.67 among males and 0.48 among females).

**Figure 7:**
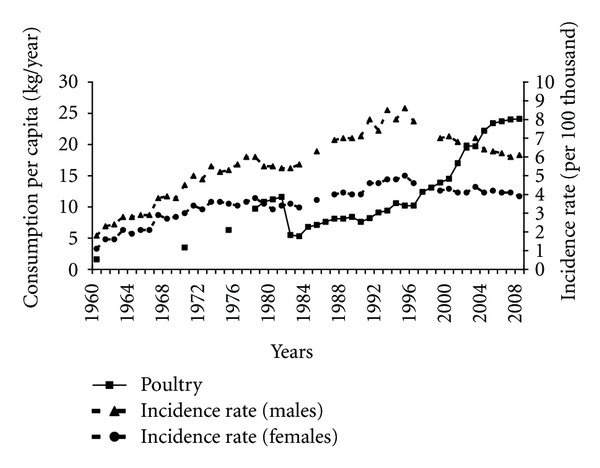
Poultry consumption and pancreatic cancer morbidity 1960–2008.

After the transformation the meat consumption structure has changed. Along with reduced red meat consumption the increase in poultry consumption took place (Figure 7). This increase negatively correlated with the pancreatic cancer morbidity (−0.88 among males and −0.57 among females). 

**Figure 8:**
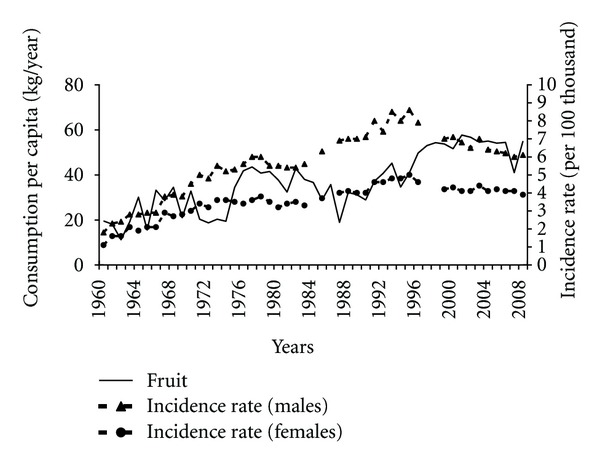
Fruit consumption and pancreatic cancer morbidity 1960–2008.

Positive trends observed in the recent years can also be a result of the higher fruit consumption compared to previous years (Figure 8). In the years 1990–2008 a high, negative correlation between analyzed variables was observed (−0.62 among males and −0.50 among females). 

**Figure 9:**
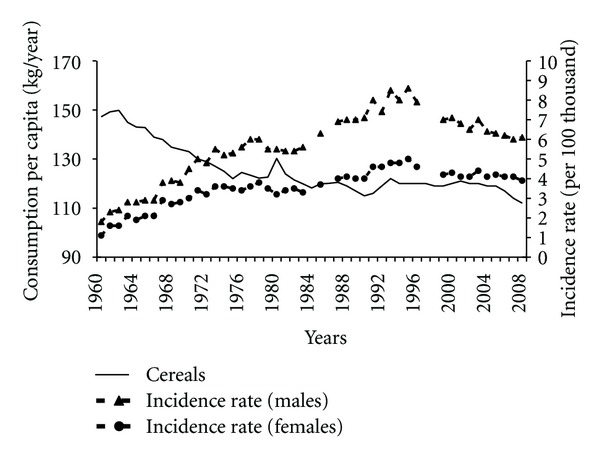
Cereals consumption and pancreatic cancer morbidity 1960–2008.

**Figure 10:**
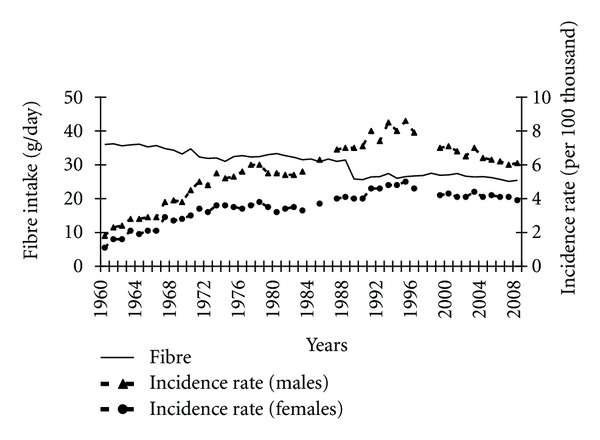
Fibre intake and pancreatic cancer morbidity 1960–2008.

A decline in the cereals consumption may have been a factor that highly contributed to the increased pancreatic cancer morbidity before 1995 (Figure 9). Almost a complete, negative correlation between these variables was observed (−0.93 among males and −0.91 among females) in the years 1960–1989. Cereals are the most important source of fibre in the typical Polish diet; therefore, fibre content in the diet also fell in this period (Figure 10). It correlated negatively with incidence rates (−0.84 among males and −0.89 among females).

**Figure 11:**
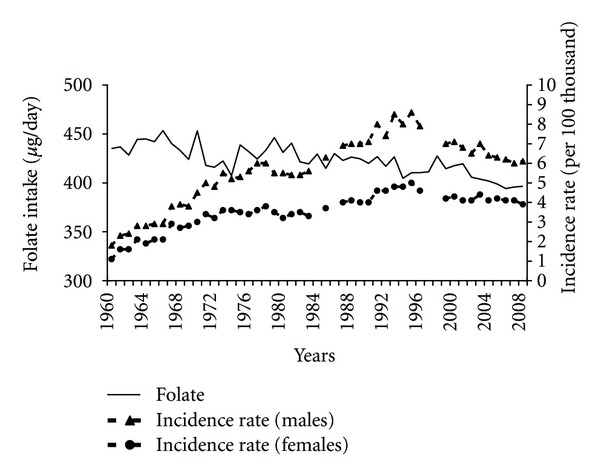
Folate intake and pancreatic cancer morbidity 1960–2008.

The consumption of products containing folate is considered as one of nutritional factors that decrease pancreatic cancer risk. Despite certain fluctuations, the folate content in a diet in the analyzed period decreased (Figure 11). In the years 1960–1989 this tendency correlated negatively with the increased pancreatic cancer morbidity (−0.45 among males and −0.49 among females).

**Figure 12:**
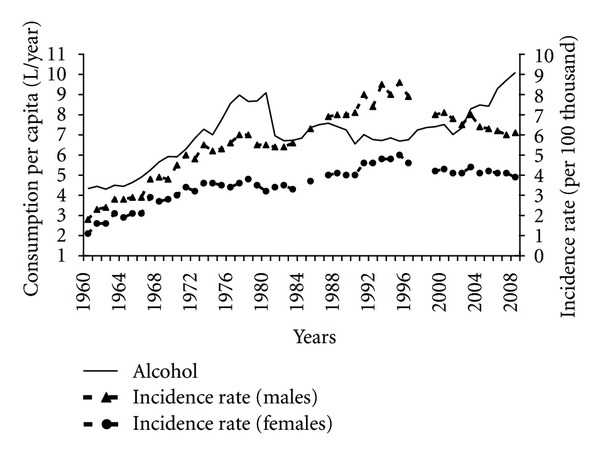
Alcohol consumption and pancreatic cancer morbidity 1960–2008.

The risk of pancreatic cancer may be indirectly boosted by alcohol as a result of chronic pancreatitis. Alcohol consumption was growing visibly between 1960 and 1980 (Figure 12). In the early 1980s its consumption fell to remain at the comparable level in later years. However, since in the early 1980s alcohol began to be rationed in Poland, there is a strong possibility that data on the consumption can be underestimated. Nevertheless, a very high positive correlation between alcohol consumption and incidence was observable in that period (0.86 among males and 0.82 among females). After 2001 another increase in alcohol consumption took place, which has not reversed a positive tendency of reduced pancreatic cancer morbidity so far.

**Figure 13:**
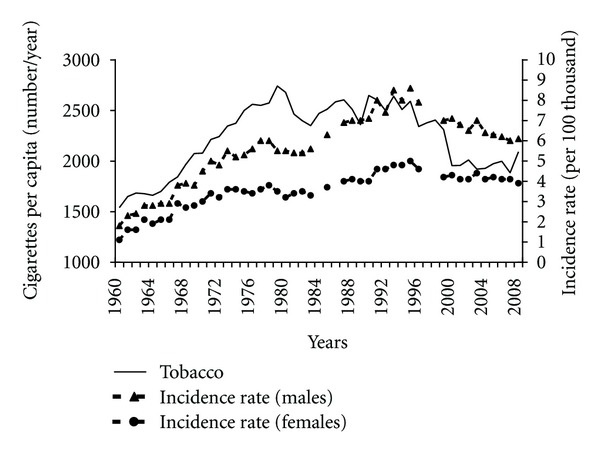
Tobacco smoking and pancreatic cancer morbidity 1960–2008.

Apart from nutrition, other environmental factors can influence the development of pancreatic cancer, among others, tobacco smoking. The number of cigarettes per person a year was increasing from 1960 to 1979, followed by downward or upward trends lasting a number of years (Figure 13). On the basis of the number of cigarettes smoked and incidence rates, a positive correlation was found in the entire period under analysis (0.55 among males and 0.44 among females). Frequent smoking could contribute to the increased incidence in the beginning. A significant decrease in cigarettes consumption in the years 1995–2000 can be one of the factors that positively influence the observed downward trend as regards pancreatic cancer morbidity. 

## 4. Discussion

It is very difficult to prove the relationship between environmental factors, lifestyle factors such as tobacco smoking, body weight, diet, and cancer. The abovementioned relationship needs to be proven on the basis of epidemiological research [16]. Moreover, the influence of various factors on carcinogenesis needs to be proven on the basis of experimental study.

The risk of pancreatic cancer is probably increased by a number of different factors. One of the best proven risk factors is tobacco smoking [1, 17]. There is not enough evidence to claim with absolute certainty that there is a relationship between cancer and diet although the negative impact of body fatness, especially abdominal fatness, excessive energy intake, high consumption of fat, red meat, and sugar is frequently taken into consideration. On the other hand, high consumption of fruit, products containing dietary fibre, folate, and other vitamins can probably reduce the risk [18].

High energy intake in Poland up to 1989 was correlated with pancreatic cancer incidence. In this case, oxygen-free radicals, which cause DNA damage to cells, play a very important role in the process of carcinogenesis [19, 20]. Excessive energy content in a diet can also lead to overweight and obesity. Studies show that over a half of adult Poles are overweight or obese, and very often this obesity is abdominal one [21, 22]. Unfortunately, no long-term, national, or systematically repeated surveys were carried out in this field in Poland, the results of which would allow an establishment of statistical correlation.

Until the transformation period, the high energy intake was related to excessive consumption of fats, cholesterol-rich products, and sugar, which evidently correlated with the growing pancreatic cancer incidence. 

There are some mechanisms that can explain an association between high fat consumption and pancreatic cancer risk. When fat gets into the duodenum, cholecystokinin is released [23]. That stimulates lipases secretion by pancreas. However, when high amounts of fatty acids found their way to duodenum for a long time, pancreatic hypertrophy or hyperplasia may take place. Then pancreas is more prone to adverse activity of carcinogens. Moreover, high content of certain fatty acids in a diet can contribute to the secretion of bile acids to the pancreatic duct [24]. Bile acids can stimulate cyclooxygenase-2 (COX-2) release and promote carcinogenesis.

Excessive energy intake and high content of saturated fat and sugar in a diet are associated with insulin resistance [25–27] and diabetes that can increase the risk of pancreatic cancer [28]. Prolonged hyperinsulinemia increases endogenous levels of insulin-like growth factor-1 (IGF-1) [29]. Both insulin and IGF-1 affect the increase in abnormal cell proliferation and reduction of apoptosis.

The influence of fat and cholesterol content in a diet on the development of pancreatic cancer is not definite. The data from a population-based case-control study conducted in California provided some evidence that fat and cholesterol may increase the risk of pancreatic cancer [30]. Some analyses indicate the adverse activity of animal fats in particular [31, 32]. On the other hand the authors of the Multiethnic Cohort Study in Hawaii found no associations of pancreatic cancer risk with intake of total fat, saturated fat, or cholesterol [33].

Results of our study demonstrate that pancreatic cancer incidence in Poland could be more linked to animal fats consumption than to total fats consumption. After 1995 the incidence fell despite the growing total fats consumption. This is related to beneficial changes in the fat consumption structure, as the share of animal fats radically fell in favour of the consumption of vegetable fats.

Surveys also confirm the adverse influence of high sugar consumption. According to the abovementioned study from Hawaii, the risk of pancreatic cancer increased with higher intake of total sugars, fructose, and sucrose [34]. Also the results of a prospective study conducted in Sweden confirmed that the high consumption of sugar and high-sugar foods might be associated with a greater risk of pancreatic cancer [35].

Another factor that can possibly contribute to the pancreatic cancer incidence is red meat. It seems that the decline in consumption of this food after 1989 had a critical significance to the downward trend. Red meat is a source of heme iron, and free iron can favour the creation of free radicals [1]. The method of preparing dishes can also be important in this respect [1, 36]. Cooking at high temperatures (frying or grilling) produces heterocyclic amines and polycyclic aromatic hydrocarbons that pose a potential risk of cancer.

Many surveys confirm the relationship between red meat consumption and increased risk of pancreatic cancer, but not all of them. Recently published results of meta-analysis of prospective studies indicated that red meat consumption was positively associated with pancreatic cancer risk in men but not in women [37]. On the other hand, according to the other meta-analysis, red meat was associated with higher pancreatic cancer risk in case-control studies but not in cohort studies [38]. Results from the large population-based prospective cohort of women in Sweden support a possible positive association of long-term red meat consumption and an inverse association of long-term poultry consumption with pancreatic cancer risk [36]. Since many surveys have not confirmed the relation between the poultry consumption and the risk of pancreatic cancer [33, 39, 40], the authors of Swedish survey believe that their observations may be attributable to chance [36]. Our analysis also attributes reduced morbidity to the increased poultry consumption; it seems though that, above all, it was a result of the changing meat consumption structure.

Fruit can protect pancreas against cancer. They are a source of vitamin C and other antioxidants; they have the ability to trap free radicals and reactive oxygen molecules, protecting against oxidation damages [1]. Moreover, flavonoids found in fruit inhibit the metabolic activation of carcinogens by cytochrome P450 enzymes or by detoxifying and cellular defensive enzymes [1, 41]. 

Some surveys confirm a positive influence of fruit as regards protection against pancreatic cancer [38, 42], and others do not observe such a link [43]. In Poland, the growth in fruit consumption may have contributed to the reduced incidence in recent years. 

Reduced risk of pancreatic cancer may be related to the consumption of fibre-rich products, including cereals, particularly wholegrain ones. Potential protective influence of fibre intake on pancreatic cancer could be explained through association with insulin resistance, triglyceride, and high density lipoprotein (HDL) level [42]. Some studies support the hypothesis that consuming more whole-grain or high-fibre foods may reduce the risk of pancreatic cancer [42, 44].

In Poland, the decline in consumption of products containing fibre, including cereals, has probably increased the risk of pancreatic cancer.

Food containing folate is one of the factors reducing the risk of pancreatic cancer. It is assumed that folate influences carcinogenesis through its role in methylation reactions, nucleotide synthesis, and DNA repair [1, 6]. The authors of the population-based prospective study in Sweden found a strong inverse association between the dietary folate intake and the risk of pancreatic cancer [45]. However, folic acid from supplements did not show a protective effect. Other surveys have also confirmed the positive impact of folate [46, 47] on the prevention of pancreatic cancer, although not all of them [48, 49].

In Poland we have observed a decreasing folate content in a diet for many years. It seems that this fact had more significance before the transformation, where other nutritional aspects also influenced the pancreatic cancer morbidity. 

Alcohol is not considered a direct risk factor for pancreatic cancer [7, 8]. However, chronic alcohol drinking can cause pancreatitis whilst heavy alcohol consumption leads to diabetes mellitus. These two diseases are risk factors for pancreatic cancer. Moreover its metabolites, such as acetaldehyde and fatty acid ethyl esters, can modify metabolic pathways involved in the inflammatory response and carcinogenesis. A hospital-based case-control study in Texas has shown that heavy alcohol consumption (>60 mL ethanol/day) significantly increased pancreatic cancer risk [50]. According to a population-based case-control study in different US towns, alcohol drinking at levels typically consumed by the general population did not appear to be a risk factor for pancreatic cancer although heavy consumption could be related to risk [51].

The consumption of alcohol in Poland was growing until 1980 and could be one of the factors favouring the pancreatic cancer morbidity. The introduction of alcohol sale rationing in the 1980s contributed to the reduced consumption however, these data may be underestimated. It is hard to definitely state that this fact stood in any relation to the future reduction in incidence rates. The recently observed increased alcohol consumption has not had a negative impact on the incidence. Taking into account the indirect influence of alcohol on pancreatic cancer, we cannot rule out that its adverse effects will manifest themselves later on. 

Tobacco smoking is the strongest environmental risk factor of pancreatic cancer causing 20–25% of all pancreatic tumours [2, 52]. It is most probably connected with the mutagenic effect of the tobacco smoke components such as heterocyclic amines and polycyclic aromatic hydrocarbons (PAH) on protooncogenes in cells, which cause especially K-ras mutations [53]. It is an important early stage of carcinogenesis. After PAH enter the organism, they are metabolized by detoxifying enzymes into forms capable of interacting with DNA. As a result of the activity of the P450 enzyme system (CYP1A and CYP1B), active epoxy compounds are formed. These are then hydrolyzed by the epoxy hydrolase into diol epoxide derivatives. They can fix with DNA (in the position of N2 glutamine), and this, in turn, can lead to P53 gene mutation. The high prevalence of K-ras mutations in smokers and drinkers with pancreatic cancer might reflect combined carcinogenic effects of tobacco and alcohol [54]. Additionally, smoking is an independent risk factor for chronic pancreatitis [7, 53]. Giving up smoking substantially reduces the future incidence of pancreatic cancer [55].

In Poland for many years tobacco smoking was increasing and then remained at a high level. That was accompanied by an increasing incidence of pancreatic cancer. Later, as the number of cigarettes smoked was falling, a substantial decrease in pancreatic cancer incidence was observed. 

On the basis of research on dietary factors, which may have an impact on pancreatic cancer morbidity in Poland, it has been proven that the majority of observations conducted in other countries also concern Poles. According to the conducted investigations, the factors that increase the risk of developing pancreatic cancer, to different extents, is high consumption of red meat, fats, especially of animal origin, sugar, and cholesterol intake, and the factor that reduces the risk is consumption of fruit, cereals, and, perhaps, also folate and dietary fibre. What is very interesting and important from the point of view of measures to prevent pancreatic cancer is the fact that the growth rate of pancreatic cancer incidence in Poland has been reversed since 1995. This state of affairs is associated with better diet, that is, lower energy intake, along with lower red meat and animal fat consumption and higher fruit consumption. It also seems that positive changes as regards smoking have had a positive impact in the subjective issue.

Pancreatic cancer is a type of cancer that has an especially poor prognosis [1, 2]. Many cases are diagnosed at a late stage of the disease, and this fact makes it impossible to implement any radical surgical treatment. Special attention should therefore be paid to prevent this type of cancer by changing lifestyle habits. As it appears from presented research, tobacco smoking and alcohol consumption should be kept low. Moreover, an educational campaign should be conducted on a wide scale in order to further improve dietary habits.

## 5. Conclusions

Increase in the pancreatic cancer morbidity in Poland in 1960–1995 was probably related to adverse dietary patterns up to 1989: high energy and cholesterol intake, decrease in fibre and folate content in a daily diet and high consumption of total fats, animal fats, sugar and alcohol, and the decrease in cereals consumption. 

Lowering red meat and animal fats consumption as well as increasing fruit consumption after economic transformation could influence incidence reduction observed in 1996–2008. 

Changes in tobacco smoking probably also affected pancreatic cancer morbidity. Growing or large number of cigarettes smoked was accompanied by a rise in incidence, whilst reduced smoking was associated with decreased morbidity. 

## Figures and Tables

**Table 1:** Correlations between dietary factors and pancreatic cancer morbidity rates, 1960–1989, 1990–2008, 1960–2008, by sex.

Factor	1960–1989		1990–2008		1960–2008	
	Men	Women	Men	Women	Men	Women
	*r* (*P* value)	*r* (*P* value)	*r* (*P* value)	*r* (*P* value)	*r* (*P* value)	*r* (*P* value)
Energy	0.60 (<0.001)	0.57 (<0.01)	−0.29 (0.263)	−0.47 (0.055)	−0.50 (<0.001)	−0.57 (<0.001)
Total edible fat	0.94 (<0.001)	0.91 (0.001)	−0.87 (<0.001)	−0.63 (0.007)	0.75 (<0.001)	0.79 (<0.001)
Animal fats	0.90 (<0.001)	0.82 (<0.001)	0.60 (0.011)	0.30 (0.267)	−0.08 (0.616)	−0.24 (0.115)
Cholesterol	0.87 (<0.001)	0.80 (<0.001)	−0.52 (0.033)	−0.62 (0.008)	0.32 (0.035)	0.24 (0.107)
Sugar	0.88 (<0.001)	0.87 (<0.001)	0.24 (0.347)	−0.01 (0.981)	0.49 (<0.001)	0.40 (0.007)
Red meat	0.34 (0.342)	0.19 (0.193)	0.67 (0.003)	0.48 (0.050)	0.14 (0.445)	−0.03 (868)
Poultry	0.40 (0.180)	0.34 (0.248)	−0.88 (<0.001)	−0.57 (0.016)	0.15 (0.437)	0.39 (0.033)
Fruit	0.50 (0.007)	0.36 (0.059)	−0.62 (0.008)	−0.50 (0.039)	0.59 (<0.001)	0.64 (<0.001)
Cereals	−0.93 (<0.001)	−0.91 (<0.001)	0.52 (0.033)	0.56 (0.021)	−0.82 (<0.001)	−0.81 (<0.001)
Dietary fibre	−0.84 (<0.001)	−0.89 (<0.001)	0.44 (0.079)	0.38 (0.136)	−0.84 (<0.001)	−0.87 (<0.001)
Folate	−0.45 (0.017)	−0.49 (0.009)	0.71 (0001)	0.36 (0.151)	−0.58 (<0.001)	−0.68 (<0.001)
Alcohol	0.86 (<0.001)	0.82 (<0.001)	−0.87 (<0.001)	−0.57 (0.017)	0.48 (<0.001)	0.51 (<0.001)
Tobacco	0.87 (<0.001)	0.82 (<0.001)	0.72 (0.001)	0.41 (0.100)	0.55 (<0.001)	0.44 (0.003)
